# A Comparison of Diagnostic Accuracy of a Rapid Antigen Detection Test in Screening for Group A Streptococcal Throat Infection Between 3- to 10-Year-Old (Children and Preadolescents) and 11- to 21-Year-Old (Adolescents)

**DOI:** 10.7759/cureus.14840

**Published:** 2021-05-04

**Authors:** Abdullah Khan, Drew Davis, Lance Brown

**Affiliations:** 1 Pediatric Emergency Medicine, Loma Linda University Medical Center, Loma Linda, USA; 2 Pediatric Emergency Medicine, Dignity Health - St. Rose Dominican Hospital, Siena Campus, Henderson, USA

**Keywords:** rapid antigen detection test, children, adolescent, pharyngitis

## Abstract

Introduction

Pharyngitis is one of the most common childhood diseases worldwide. We intended to compare the performance of one such rapid antigen detection test (RADT) using lateral flow immunoassay technique, between 3- to 10-year-old (children and preadolescent) and 11- to 21-year-old (adolescents).

Methods

Children and adolescents attending the pediatric ED with complaints of throat pain and signs of pharyngeal and tonsillar inflammation were tested by both the RADT and throat culture (TC) directed towards group A streptococcal (GAS) between April and June of 2016. The prevalence, sensitivity (SN), specificity (SP), positive predictive value (PPV) and negative predictive value (NPV) were calculated against throat culture, the gold standard for the diagnosis of GAS pharyngitis. Comparisons between the two age groups were made using the Chi-square test

Results

Of 202 patients, 123 (61%) patients were between 3-11 and 79 (39%) between 11-21 years of age. A positive throat culture was recorded in 56 patients yielding an overall prevalence of GAS pharyngitis at 28%. For the whole sample, the screening RADT had an SN, SP, PPV and NPV of 79%, 90%, 75%, and 92%, respectively. Also, there was no statistically significant difference between the two groups in terms of SN, SP, PPV and NPV.

Conclusion

The RADT in use at our institution, performed comparable to studies reported in the literature using a similar technique in both preadolescent and adolescent age groups.

## Introduction

Sore throat is a common presentation in pediatric office settings and emergency departments [1]. Most cases are caused by a viral infection [2]. Strep throat, a bacterial infection, is the cause in about 25% of children [2]. The management of Group A streptococcal (GAS) pharyngitis in children alone costs between $224 and $539 million per year [3]. The Infectious Diseases Society of America (IDSA) recommends laboratory testing with a rapid antigen detection test (RADT) and/or throat culture (TC) to distinguish viral pharyngitis from GAS pharyngitis [4].

The RADTs have different sensitivities and specificities in the pediatric population depending on the type of techniques used [5]. In our institution we use immunochromatographic lateral flow immunoassay, to screen GAS pharyngitis. We used the same technique in our study.

In our literature review, we were able to find only one study that compared performance of RADT in different age groups [6]. This study was conducted in Turkey. Our objective was to evaluate the performance of RADT (lateral flow immunoassay) in the pediatric age group and compare the diagnostic accuracy between preadolescents (3-10 years) and adolescents (11-21 years).

## Materials and methods

The study was conducted in the urban community pediatric emergency department (PED). Our study is IRB approved. We enrolled children presenting to PED over a period of three months from April 2016 to June 2016. Our inclusion criteria were: (1) children and adolescents between the ages of 3 and 21 years, (2) presenting with complaint of throat pain and signs of pharyngeal inflammation (erythema, tonsillar and pharyngeal swellings with or without exudates). We used the definition of pre-adolescents and adolescents as outlined by the American Academy of Pediatrics [7].

We used RADT directed towards GAS infection utilizing enzyme immunoassay (lateral flow/immunochromatographic assays) technique for the study. For all patients, we used two throat swabs to collect a throat sample. After the swabs were removed from the sterile packaging, they were held by the handle without touching the tip of the swabs. Once the patient opened the mouth, the tongue depressor was used to press the tongue against the floor of the mouth. The swabs were then rubbed against the tonsillar and pharyngeal surfaces and removed without touching other areas of the mouth. One swab was used to run RADT in the emergency department according to the manufacturer’s instructions [8]. The RADT tests were conducted and analyzed by resident physicians under direct supervision of attending physicians. The other swab was sent to the laboratory for throat culture in a sterile container. The throat culture was performed on all children irrespective of the positive or negative results of the RADT.

We used 2x2 tables to calculate sensitivity (SN), specificity (SP), positive predictive value (PPV) and negative predictive value (NPV), for the whole sample (children between 3 and 21 years). Then we divided the sample into two groups, children and preadolescent (3-10 years) and adolescents (11-21 years). We defined the adolescent age group from 11 to 21 years based on the American Academy of Pediatrics guidelines [7]. We re-calculated the SN, SP, PPV, NPV for both groups. The p values for comparison were calculated using chi-square test. We calculated medians for continuous variables and 95% confidence intervals (95 CI) for proportions.

## Results

**Figure 1:**
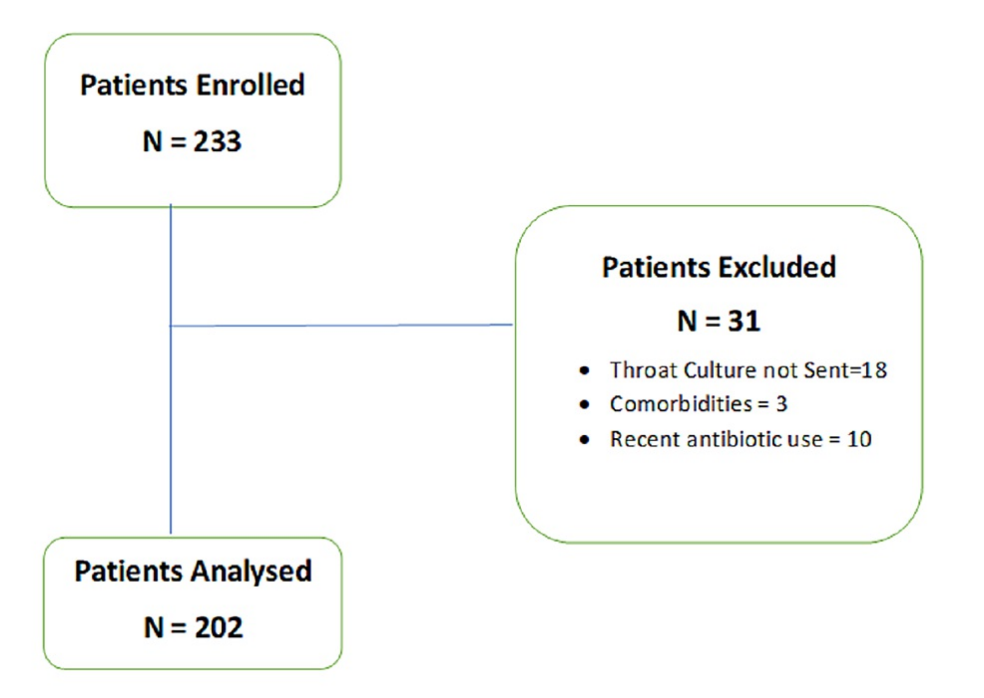
Summary of patients enrolled with pharyngitis and reasons for exclusion.

In this study, we enrolled 233 patients. Out of these, three patients with comorbidities (asthma, sickle cell disease), 10 with recent antibiotic use (defined as use of antibiotics over four weeks before presentation) and 18 with no throat cultures were excluded (Figure 1).

The median age for the whole sample was nine years (IQR 5-15). Out of these 88 (44% 95 CI 37-51) were male and 114 (56%, 95 CI 49-63) were female. The prevalence of GAS pharyngitis was 28% in the whole sample. For the whole sample, RADT performed with sensitivity and specificity of 79 (95 CI 66-88) and 90 (95 CI, 84-94), respectively. The PPV and NPV were 75 (95 CI 64-83) and 92 (95 CI 81-91) (Table 1).

**Table 1:** Sensitivity, specificity, positive predictive value, and negative predictive value of all patients included in the analysis (n = 202).

	Total patients between 3 and 21 years of age % (95% CI)
Sensitivity (SN)	79 (66-88)
Specificity (SP)	90 (84-94)
Positive predictive value (PPV)	75 (64-83)
Negative predictive value (NPV)	92 (81-91).

Also, RADT performed similarly when compared between preadolescents (Group A) and adolescents (Group B). We had 123 children and preadolescents (61% 95 CI 54-68) in Group A and 79 adolescents (39% 95 CI 32-46) in Group B. The median age of Group A was six years (IQR 5-8). Out of these, 59 (48% 95 CI 39-57) were male and 64 (52% 95 CI 43-61) were female. The median age of Group B was 17 years (IQR 14-19). Out of these, 29 (37% 95 CI 26-48) were male and 50 (63% 95 CI 52-74) were female. The prevalence was 26% in Group A and 30% in Group B. The sensitivity in Group A was 81 (95 CI 64-93) compared to 75 (95 CI 53-90) in Group B (p-value: 0.30). The specificity of RADT was recorded as 89 (95 CI 81-95) in Group A and 91 (95 CI 80-97) in Group B (p-value: 0.64) (Table 2).

**Table 2:** Comparison of performance of rapid antigen detection test against gold standard (throat culture) between Group A (children and preadolescents) and Group B (adolescents).

	Patients between 3 and 10 years of age (Group A) % (95% CI)	Patients between 11 and 21 years of age (Group B) % (95% CI)	p values
Sensitivity (SN)	81 (64-93)	75 (53-90)	p=0.30
Specificity (SP)	89 (81-95)	91 (80-97)	p=0.64
Positive predictive value (PPV)	72 (59-83)	78 (60-90)	p=0.34
Negative predictive value (NPV)	93 (87-97)	89 (81-94)	p=0.32

The PPV values were 72 (95 CI 59-83) for Group A and 78 (95 CI 60-90) for Group B with p-value = 0.34. Similarly, the NPV for Group A and Group B were 93 (95 CI 87-97) and 89 (95 CI 81-94), respectively, with p-value = 0.32 (Table 2).

## Discussion

The Infectious Diseases Society of America and the European Society for Clinical Microbiology and Infectious Diseases have incorporated RADTs in clinical practice guidelines [4]. These guidelines suggest to send throat cultures in cases of negative RADT but suggest not to send throat cultures as backup for positive RADT due to high specificity [4]. Different RADT methods in use can be classified into three categories: (1) enzyme immunoassay (lateral flow/immunochromatographic assays), (2) optical immunoassays (OIAs) and (3) newer molecular-based techniques, such as polymerase chain reaction (PCR), and fluorescence in situ hybridization (FISH) [9]. Lateral flow immunoassays are a type of first-generation immunoassay technique that utilizes a paper strip coated with antigen-specific antibiotics. As the sample is added the antigen flows tangentially across the paper strip, reacts with antibody, and produces a color line [10]. OIAs are second-generation immunoassays that utilize the reflective surface coated with film that attenuates certain wavelengths giving it a gold color. As the antigen bind to the film, it changes the film and attenuates different wavelengths creating a purple color (positive test result) [10]. FISH technique uses “DNA fragments incorporated with fluorophore-coupled nucleotides as probes to examine the presence or absence of complementary sequences in fixed cells or tissues under a fluorescent microscope” [11]. The PCR is an enzymatic assay that involves amplification of the target antigen DNA [12].

The bacterial swab of throat culture is the gold standard in diagnosing Group A streptococcal infection [13]. However, there is long lag time between the collection of the specimen and results. On the contrary, RADTs provide results within a few minutes [14]. All of these RADT techniques have different sensitivities reported. For lateral flow immunoassay sensitivities have been reported as low as 66% and 70% in studies by Limbergen et al. and Gurol et al. respectively [15,16]. Whereas Tanz et al. and Cohen et al. reported sensitivities of 70% and 85% respectively for lateral flow immunoassay [5,9,17]. All these studies reported high specificities between 95% and 99% for lateral flow immunoassay [5,9,15,17]. For OIAs, the results are also reported variably across different studies, Kuhn et al. reported sensitivity and specificity of 89% and 96%, respectively [18]. On the contrary, in a study by Gieseker et al., the lateral flow immunoassay performed better than OIA with sensitivities of 93% and 75 % and specificities of 92% and 97%, respectively [19]. In a meta-analysis by Lean et al. on pediatric population, the sensitivity of lateral flow immunochromatographic studies and OIA were similar at 85%, whereas the specificity of lateral flow assay was slightly higher than OIA [5]. The newer molecular techniques like PCR have sensitivities of 93% and 95% reported by Lean et al. and Rao et al., respectively [5,20]. In our study, lateral flow immunoassay performed better than Limbergen et al. and Gurol et al. except for PPV in our study was lower than Gurol et al., but sensitivities and specificities in our study were comparable to Cohen et al. and Lean et al. for both preadolescents and adolescents [5,9,15,16].

There is limited data on the comparison of performance of RADT in different age groups. According to Camurdan et al., the RADT performed similarly in all age groups [6]. Our results also suggest that RADT performed similarly in both pre-adolescents and adolescents, indicating that sensitivity of RADT is not affected by different age groups. It has been studied previously that the sensitivity varies between different settings depending on the personnel who perform the test and bacterial inoculum size. In a study by Cohen et al., the sensitivity of the RADT decreased to 40% with light inoculum of bacterial antigen in the collected sample [21]. In a study by Fox et al., the sensitivities also varied depending on the person performing the swab. They showed that test performed by trained laboratory personnel had higher sensitivity compared to non-laboratory personnel [22].

In our review of literature, it is apparent that new molecular techniques performed better than immunoassays [5,20]. With the high sensitivities of molecular techniques, the utility of back-up throat culture in cases of negative RADT becomes questionable. The current practice of sending throat culture in cases of negative RADT costs approximately 8 million dollars to prevent one case of rheumatic heart disease [23]. The drawback of new molecular screening tests is delayed turnaround compared to lateral flow immunoassay which can be ready in few minutes [14]. Therefore RADT based on lateral flow immunoassays are still widely used. There is room for further studies to examine different techniques to improve sensitivities of lateral flow immunoassays and cost-effective analysis of different RADT techniques with throat cultures.

One of the limitations of the techniques utilized to identify GAS is the inability to differentiate between true infection and carrier state. One way to identify if the episode is a true GAS infection is by obtaining blood specimens to identify antibody titers against GAS [24]. The other way is to obtain a repeat throat culture once the patient becomes asymptomatic. If the repeat culture is negative, then the episode was a true GAS infection [25]. Both these methods are not practical in emergency department settings. In clinical setting, a physician can have an approximation of the probability of true infection based on symptomatology. The children with true streptococcal pharyngitis are likely to have symptoms of fever, tender anterior cervical adenopathy, exudative pharyngitis and palatal petechiae and show resolution of symptoms within one to two days after start of antibiotics [26].

Our study has several limitations. First it is a single-center study. Second, we have a small sample size. Third, we did not use newer, especially molecular techniques of RADT. Finally, Group A streptococcus carriers were not evaluated in our study. Further studies are needed to evaluate the diagnostic accuracy of RADT in patients who are not streptococcal carriers.

## Conclusions

We conclude that immunochromatographic lateral flow immunoassay for detection of group A beta-hemolytic streptococcus has lower diagnostic accuracy than newer techniques. The diagnostic accuracy of RADT in both preadolescents and adolescent groups was similar. 
